# Supplementary material for: Large-scale group-hierarchical DEMATEL method for complex systems
Source: PLoS One. 2023 Dec 4;18(12):e0288326. doi: 10.1371/journal.pone.0288326 (PMC10695399; doi:10.1371/journal.pone.0288326)
Supplement: Attachment — Submitted filename: Comments about PONE-D-23-09228.docx [file pone.0288326.s001.docx]

**Comments about PONE-D-23-09228**

The manuscript considers the existence of hierarchy with numerous system factors in complex systems, and proposes a hierarchical DEMATEL method for large-scale group decision-making to make DEMATEL better suited for the identification of critical factors in complex systems. Then it is applied to identify and analyze the key factors that influence combat capability, which is a typical complex system, and explain the superiority of the proposed method through comparative analysis. There are certain innovative points in this manuscript. However, some theoretical errors occurred in some places and there are still several problems that need to be explained or modified.

1. The definition of horizontal decomposition and vertical decomposition in Line 229 need to be explained briefly, and the case used in this manuscript does not involve the horizontal decomposition part, so it is recommended to reconsider whether to keep this part.
2. The combat capability system shown in Fig 5 is a three-level structure according to the theory of Yuanwei Du’s paper. Why is the manuscript explained as a two-level structure in Line 452?
3. Also in Fig 5, $F_{3\supset1}$、$F_{3\supset2}$ should be modified to $F_{2\supset1}$、$F_{2\supset2}$ since the system $F$only has two subsystems in level 1.
4. As can be seen from Table 4, the weight of expert 1 is 0.0573 when judging the degree of the influence on the system $F_{1\supset1}$ itself. Why does the weight change to 0.054 in Fig 8?
5. There may be a misunderstanding of “based on which 20% of these experts' decision data will be randomly perturbed” in L605-606. What does “these experts” refer to? “20% of these experts' decision data will be randomly perturbed” means that the data of 30% of the experts who have been disturbed will be perturbed or means that the data of all the experts involved in scoring will be perturbed?
6. In the second row of Table 9, when $p_{1}=0.04,p_{2}=0.06$, the indicators of Method 1 and Method 2 are significantly different from other situations, and it is recommended to provide a reasonable explanation.
7. The conclusion section should add future research work.
8. English language of this manuscript should be improved. Please carefully proofread your manuscript to improve its presentation and readability. For instance, the phrase “For example” appears multiple times, with two consecutive lines appearing in Line 287 and Line 288.

Besides, some format errors should be corrected in this manuscript.

1. The first line of each paragraph should be indented, while Line 433、Line 440 and Line 487 do not.
2. Tables 6、7、8 are not three-line tables.
3. Definition 1 in Line 552 and Definition 2 in Line 567 are not bolded while others are bolded. Please keep consistence.
4. The subheading 4.2.2 indents while others do not.
5. The right border of the 10th group in three-line table of Table 9 needs to be removed.
6. The references contain an excessive number of formatting errors. For example, references [21]、 [22]and [36].
