# Supplementary material for: Large-scale group-hierarchical DEMATEL method for complex systems
Source: PLoS One. 2023 Dec 4;18(12):e0288326. doi: 10.1371/journal.pone.0288326 (PMC10695399; doi:10.1371/journal.pone.0288326)
Supplement: Attachment — Submitted filename: PONE-D-23-09228R1 review.docx [file pone.0288326.s003.docx]

The revised manuscript improved a lot both in writing and formatting. However, there are still several problems that need to be explained or modified.

1. Table 1 is not three-line table.
2. English language should be improved. For instance, the phrase “In summary” appears in two consecutive paragraphs in Line 99 and Line 103.
3. The understanding of vertical decomposition and horizontal decomposition in this manuscript is different from Du’s paper which you refer to. The manuscript argues that “Vertical decomposition refers to splitting a complex function into multiple single and simple functions based on functional attributes. Horizontal decomposition refers to hierarchically dividing a system according to its hierarchical characteristics”. However, Du points that horizontal decomposition focuses on dividing the critical factor identification problem of complex systems into several simple problems and vertical decomposition focuses on dividing the complex system into multi-level subsystems under a specific rule. Horizontal decomposition provides the rules for making vertical decomposition.
4. In line 337, the manuscript uses $F_{1\supset2}$ in Fig.2 to explain the subscript, but Fig.2 does not include $F_{1\supset2}$.
5. There is a doubt about the combat capability system shown in Fig.6 is a three-level structure. The first level are $F_{1}$ and $F_{2}$, the second level are the 5 subsystems of communication, intelligence, command, logistics and fire support, and the third level are the factors $f_{i}\in\left\{f_{1},f_{2},\cdot\cdot\cdot,f_{16}\right\}$, which is a component of *F* and cannot be decomposed.
