# Supplementary material for: Large-scale group-hierarchical DEMATEL method for complex systems
Source: PLoS One. 2023 Dec 4;18(12):e0288326. doi: 10.1371/journal.pone.0288326 (PMC10695399; doi:10.1371/journal.pone.0288326)
Supplement: Attachment — Submitted filename: Response to Reviewers.docx [file pone.0288326.s004.docx]

**Response to Comments**

We would like to express our gratitude to the reviewers and editors for their time and effort in reviewing the manuscript. We have carefully considered all the comments and revised the manuscript accordingly. The major changes can be found in the revised version titled “**Revised Manuscript with Track Changes**”. In the following section, we provide detailed responses to each comment made by the reviewers.

**Response to Reviewer 4**

The revised manuscript improved a lot both in writing and formatting. However, there are still several problems that need to be explained or modified.

**Response:**

Thank you for your recognition of our manuscript revision work. With regard to the issues you mentioned this time, we reply to each of them as follows.

**Comment 1:**

Table 1 is not three-line table.

**Response 1:**

Thank you for your reminder. We have revised Table 1 and the sections that span two pages have been dealt with by way of a continuation table.

**Comment 2:**

English language should be improved. For instance, the phrase “In summary” appears in two consecutive paragraphs in Line 99 and Line 103.

**Response 2:**

Thank you for your suggestion. There was a problem with semantic repetition in the original formulation in Line 99 and Line 103, and we have redacted the repetition. Thank you for identifying this problem.

**Comment 3:**

The understanding of vertical decomposition and horizontal decomposition in this manuscript is different from Du’s paper which you refer to. The manuscript argues that “Vertical decomposition refers to splitting a complex function into multiple single and simple functions based on functional attributes. Horizontal decomposition refers to hierarchically dividing a system according to its hierarchical characteristics”. However, Du points that horizontal decomposition focuses on dividing the critical factor identification problem of complex systems into several simple problems and vertical decomposition focuses on dividing the complex system into multi-level subsystems under a specific rule. Horizontal decomposition provides the rules for making vertical decomposition.

**Response 3:**

Thank you for your careful analysis. In order to avoid misunderstandings to the reader and to respect the original meaning of the references made, we have modified the discussion of vertical and horizontal decomposition in the text according to the meaning given in the references.

**Comment 4:**

In line 337, the manuscript uses $F_{1\supset2}$ in Fig.2 to explain the subscript, but Fig.2 does not include$F_{1\supset2}$.

**Response 4:**

Thank you very much for pointing this out. Our intention was to use $F_{1\supset2}$ to illustrate Figure 2, but it was an oversight on our part that led to this error.

In the revised version, we have changed $F_{1\supset2}$ to $F_{1\supset1}$ to illustrate Figure 2.

**Comment 5:**

There is a doubt about the combat capability system shown in Fig.6 is a three-level structure. The first level are $F_{1}$ and$F_{2}$, the second level are the 5 subsystems of communication, intelligence, command, logistics and fire support, and the third level are the factors$f_{i}\in\left\{f_{1},f_{2},\cdot\cdot\cdot,f_{16}\right\}$, which is a component of *F* and cannot be decomposed.

**Response 5:**

Thank you for your statement and comments. Our views are in agreement with yours. Figure 6 represents the secondary structure.

You commented on this issue during the first revision and we responded with the following:

As shown in the figure below, in Yuanwei Du's paper case, the highest-level was not counted as a layer, so it is the same as our article, the combat capability system should be a two-layer structure.

We have therefore clarified this in the revised draft at the time of the first rework, and in our discussion of Figure 6, the discussion reads (Line 504 to Line 508 in the original).

The combat capability system is decomposed into a two-level structure according to the hierarchy, with the first level containing two subsystems, command and control and communications $F_{1}$, and firepower and logistical support$F_{2}$. The second level contains 5 subsystems of communication $F_{1\supset1}$, intelligence $F_{1\supset2}$, command $F_{1\supset3}$, logistics $F_{2\supset1}$, and fire support $F_{2\supset2}$. These five subsystems specifically contain these 16 specific factors.

Therefore, the text is consistent with your view. However, Figure 6 does look like a Three-level structure and has been redrawn for a clearer representation, with the parts of the presentation hierarchy highlighted to avoid misleading the reader. The revised Figure 6 is shown below:

**Fig. 6.** Hierarchy of combat capability
